# High-Efficiency Triple-Junction Polymer Solar Cell: A Theoretical Approach

**DOI:** 10.3390/molecules29225370

**Published:** 2024-11-14

**Authors:** Fazli Sattar, Xiaozhuang Zhou, Zakir Ullah

**Affiliations:** 1 Yangtze Delta Region Institute (Huzhou), University of Electronic Science and Technology of China, Huzhou 313001, China; 2 Institut de Ciència de Materials de Barcelona (ICMAB-CSIC), Consejo Superior de Investigaciones Científicas, Campus Universitari de Bellaterra, 08193 Cerdanyola del Vallès, Spain

**Keywords:** solar cell, DFT, TD-DFT, UV-Vis, HOMO, LUMO

## Abstract

This study presents the theoretical design and evaluation of a triple-junction polymer solar cell architecture, incorporating oligomers of PDCBT, PPDT2FBT, and PDPP3T as donor materials and PC_71_BM as the electron acceptor. Using density functional theory (DFT) simulations and time-dependent DFT (TD-DFT) methods, the investigation covers essential photovoltaic parameters, including molecular geometries, UV-Vis spectra, and charge transport properties. The device is structured to maximize solar energy absorption across the spectrum, featuring front, middle, and back junctions with band gaps of 1.9 eV, 1.63 eV, and 1.33 eV, respectively. Each layer targets different regions of the solar spectrum, optimizing light harvesting and charge separation. This innovative multi-junction design offers a promising pathway to enhanced power conversion efficiencies in polymer solar cells, advancing the integration of renewable energy technologies.

## 1. Introduction

The bulk heterojunction structure, which consists of binary components (an electron donor and an electron acceptor), is the most commonly used and efficient design for organic solar cells [1,2,3,4]. However, due to the narrow absorption spectrum of organic semiconductors, binary active layers can only absorb a limited portion of the solar spectrum, which hinders further efficiency improvements in single-junction organic solar cells (OSCs) [5,6,7]. To address this limitation, tandem and ternary devices have been developed. Ternary organic solar cells (Ternary OSCs) offer the simplicity of single-layer fabrication while overcoming the bottlenecks in photovoltaic technology [8,9]. In the ternary OSC architecture, the introduction of a third component can enhance light harvesting and play a critical role in exciton dissociation, charge transport, and improving the film morphology. By integrating different third components such as conjugated polymers, small molecules [10,11], and quantum dots, the primary parameters of OSCs, the open-circuit voltage (Voc), short-circuit current density (Jsc), and fill factor (FF), can be optimized individually or simultaneously [12]. Recently, ternary OSCs have achieved power conversion efficiencies (PCEs) exceeding 11% [13]. Already, fullerene subsidiaries, for example, PC_61_BM or PC_71_BM, have been taken advantage of as the overwhelm electron acceptors in OSCs, and a large portion of exploration on ternary OSCs depends on these fullerene acceptors [14,15,16,17].

The inclusion of the third component in a ternary OSC serves multiple roles, influencing both the electronic properties and the morphology of the active layer [18]. A third component with an absorption spectrum that complements the donor–acceptor blend can significantly improve the active layer’s ability to harvest light. Additionally, the third component can help optimize the blend morphology, enhancing the charge generation and transport within the device. In some cases, the third component prevents the aggregation of active materials, contributing to morphological stability over time. There are primarily three working mechanisms that can control the photovoltaic process in ternary OSCs: the (i) charge transfer, (ii) energy transfer, and (iii) parallel-linkage transfer. This is because of the different energy properties of the ternary components and various resulting morphology features having an impact on the photovoltaic process. These mechanisms are often interconnected rather than functioning independently. The key role of the third component is typically to enhance light absorption, which increases the Jsc by enabling more efficient photon capture. By carefully selecting a third component with strong absorption properties that complement the donor–acceptor blend, the overall absorption range of the active layer can be expanded.

In 2016, Jenekhe et al. [19]. reported the first fullerene-free ternary OSCs using two donors, PSEHTT and PBDTT-EFT (also known as PTB7-Th), and a novel electron acceptor, DBFI-EDOT. The incorporation of the third component led to an improvement in PCE from 8.1% to 8.52%, primarily due to the increase in Jsc from 13.82 to 15.67 mA/cm^2^, driven by the wider light-harvesting ability of PTB7-Th. Conjugated organic polymers (COPs) are being increasingly recognized as promising materials for solar cells due to their unique properties and benefits in renewable energy applications. Four prominent generations of COPs are being explored by scientists; each generation represents an advancement in the technology aimed at improving the cost-effectiveness, environmental impact, and efficiency of solar cells, making COPs a key area of research in the transition towards renewable energy sources. COPs used in polymer solar cells (PSCs) feature a tuneable band gap, allowing for precise adjustments of the band gap, HOMO, and LUMO energy levels during synthesis. These modifications directly influence the open-circuit voltage, charge transport efficiency, and short-circuit current density in PSCs.

This study aims to theoretically investigate the properties of three donor polymers—PDCBT, PPDT2FBT, and PDPP3T—paired with the fullerene derivative PC71BM as the electron acceptor. By employing oligomer models to analyze their optical and electronic properties, we seek to create low-bandgap polymers with optimized band alignment and absorption characteristics. This theoretical exploration will provide insights into how the inclusion of these polymer systems in ternary OSCs can enhance light absorption, charge transport, and the overall device performance, ultimately guiding future experimental efforts toward more efficient polymer solar cells with minimal synthetic effort.

Hence, this study focuses on the theoretical aspect of optimizing polymer donors and their interaction with the fullerene acceptor, positioning the work as a foundational guide for experimentalists.

## 2. Computational Methodology

Density functional theory (DFT) simulations at the B3LYP level of theory and 6-31G(d) basis set [20,21,22] for PC_71_BM and nth oligomers (where *n* = 1, 2, 3, 4) of PDCBT, PPDT2FBT, and PDPP3T were carried out using Gaussian 16, suit of ab ignition program, while visualizations were accomplished on Gabedit and GaussView 6.01. To reduce the computational time, the molecular structure of all these polymers was simplified by replacing the aliphatic chains that do not contribute to the π-conjugated backbone with methyl groups. It was confirmed that these alkyl chains do not affect the electronic properties of these oligomers. First, the oligomeric chain length of these polymers was optimized, and it was found that the oligomers, containing ~16 conjugated rings, can accurately represent their polymeric characteristics. Moreover, the oligomeric properties were extrapolated to infinite polymers using 2nd-order polynomial fit equation (Table S1). In order to understand the charge transferring phenomena, PC_71_BM was interacted near the backbone of these optimized oligomers. As we explained in our previous work, TD-DFT is optimistic between semi-empirical and wave function approaches regarding computational and accuracy point of view [23,24,25]. TD-DFT calculations can incorporate environmental effects and quickly give best quantitative fit to UV-Vis spectra (excitation energy) of these species, especially using hybrid functionals (B3LYP) [25,26,27]. In the case of approximate DFT, negative orbital energies (HOMO and LUMO) do not give accurate ionization potentials (IP) and electron affinities (EA), but the deviation is about 1 eV. Since the error is method-dependent and consistent for all oligomers, orbital energies can be still used to examine trends consistently [28,29,30,31,32,33,34,35,36,37,38,39,40]. Our simulated results have also a nice correlation with the experimental data, as can be seen from Table S1, so that is why the current level of theory is employed for the rest of simulations [41,42].

The photovoltaic parameters, such as reorganization energy (λ), UV-Vis spectra, and exciton binding energy (E_b_), are also simulated with the said level of theory. The stability, perturbation in electroactivity, and conductivity upon mixing of donor and acceptor species are estimated from the energy of HOMO, LUMO, and band gap. The electrons and holes carrying nature are simulated from the reorganization energy and also from the contours of HOMO and LUMO, respectively. UV-Vis and an optical gap of these polymers and their PC_71_BM-interacted systems are simulated, using TD-B3LYP/6-31G(d) level of theory.

## 3. Results and Discussions

### 3.1. Optimized Geometries

**Figure 1:**
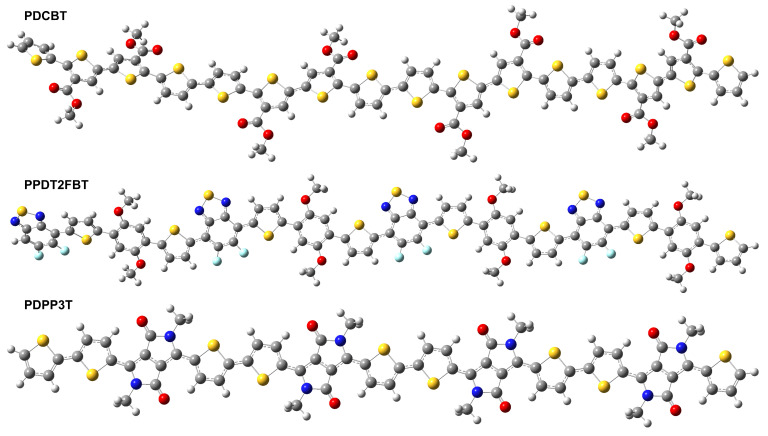
Optimized geometries of PDCBT, PPDT2FBT, and PDPP3T.

Planarity in molecular geometry and the corresponding π-electron conjugation over the backbone play an important role in the visible light absorption of a chemical substance because it allows for extended π-conjugation, which improves the overlap of molecular orbitals along the backbone. This can result in better electronic conductivity and more efficient light absorption, making these materials useful for applications such as organic solar cells. The optimized geometric structure of these polymers (shown in Figure 1) maintains a planar structure. Due to planarity, it indicates strong π-conjugation, which would enhance the absorption of visible light due to effective electron delocalization. Due to steric hindrance, the side chains in polymers like PPDT2FBT and PDPP3T were not introduced, which significantly influenced their structural and electronic properties. Hence, planarity makes the π-conjugation which is helpful for optical properties. The optimized molecular structure of PDCBT, as shown in Figure 1 (PDCBT), exhibits good planarity due to its conjugated backbone, contributing to strong π-electron delocalization and efficient light absorption that has no twists and deviation from planarity. PPDT2FBT likely shows significant π-conjugation as well, while the presence of fluorine atoms may introduce additional electronic effects, such as lowering the LUMO energy and modifying the band gap. This could enhance its electron-accepting properties in polymer solar cells, as shown in Figure 1 (PPDT2FBT), whereas, in Figure 1 (PDPP3T), the PDPP3T polymer exhibits strong π–π stacking interactions due to its structural features and shows excellent charge transport properties, making it suitable for OSCs.

### 3.2. UV-Vis Spectral Analysis of PDCBT, PPDT2FBT, and PDPP3T

**Figure 2:**
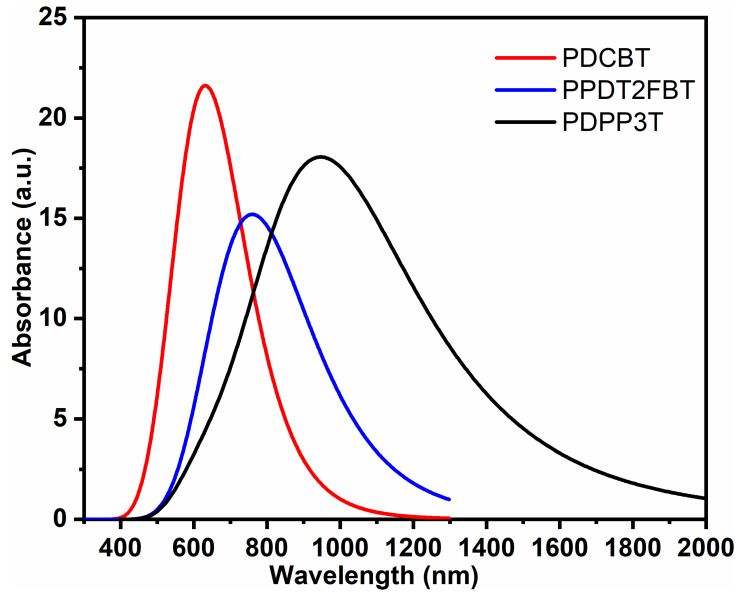
UV-visible absorption spectra of PDCBT, PPDT2FBT, and PDPP3T.

Theoretically, the electronic absorption spectra of PDCBT, PPDT2FBT, and PDPP3T at TD-DFT provide insights into the visible light absorption characteristics of the polymers, with particular focus on the key absorption peaks as in Figure 2. For PDCBT, the simulated absorption spectrum shows a significant peak at 690 nm, with an oscillating strength of five. This peak corresponds to the electronic transition from the HOMO to the LUMO. This theoretical value compares with an experimental absorption peak recorded at 650 nm [43]. For PPDT2FBT, two major peaks are observed in the simulated absorption spectrum. The first peak occurs at 440 nm with an oscillating strength of 2.5, while a second peak is observed at 755 nm with an oscillating strength of 3.4. The experimental optical absorption of PPDT2FBT in a thin film shows a peak at 880 nm, with a λ_max_ at 789 nm [44]. The observed discrepancy between the theoretical and experimental data is due to the differences in the environment (solution versus solid-state), as well as the effect of polymer packing in thin films. For PDPP3T, the theoretical UV-Vis spectrum shows an absorption peak at 990 nm, with an oscillating strength of 4.5. The polymer absorbs light in the range of 650–950 nm, with the λ_max_ recorded at 795 nm in the solution state [45]. Similar to PPDT2FBT, the theoretical red shift is ascribed to the gas-phase simulation.

### 3.3. Theoretical vs. Experimental Data for Polymer Complexes

**Figure 3:**
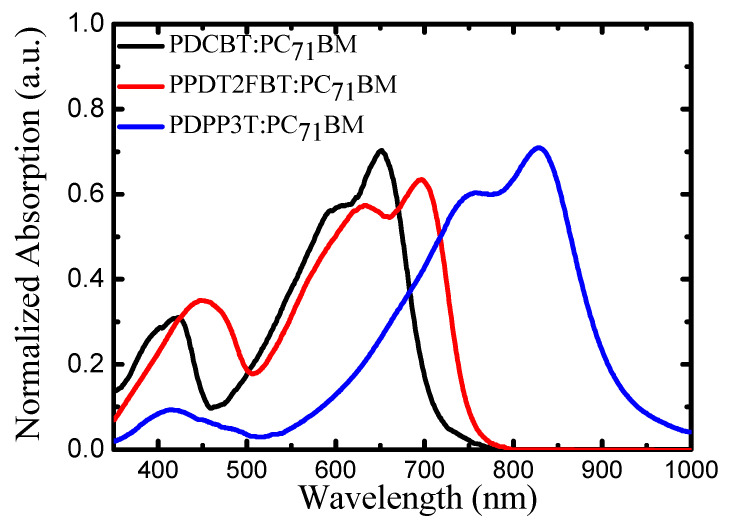
Experimental UV-Vis spectra of PDCBT-PC_71_BM, PPDT2FBT-PC_71_BM, and PDPP3T-PC_71_BM Complexes.

The theoretical UV-Vis absorption spectra of the PDCBT-PC_71_BM, PPDT2FBT-PC_71_BM, and PDPP3T-PC_71_BM complexes were simulated and compared with the experimental spectra, as depicted in Figure 3 (PDCBT-PC_71_BM, PPDT2FBT-PC_71_BM, and PDPP3T-PC_71_BM (1:2, *v*/*v*)), prepared from a processed 1,8–diiodooctane solution. The UV-Vis spectra in solution were recorded on a microplate reader (BioTek SYNERGY H4) at a concentration of 50 μM of each probe in 1,8–diiodooctane. Generally, light absorption by conjugated polymers results in strong absorption in the visible region, where electrons are transferred from the HOMO to the LUMO. The absorption spectra for these polymers show significant red-shifting with the elongation of the conjugated backbone, a trend that correlates with the effective π-conjugation in these systems.

The PDCBT-PC_71_BM complex displays a strong absorption peak at 643 nm, corresponding to the first allowed π → π* transition, capable of red-light absorption. This peak plays an important role in capturing sunlight within the red region of the solar spectrum. For the PPDT2FBT-PC_71_BM complex, a red-shifted absorption peak is observed at 772 nm, which suggests strong electronic coupling between the polymer and fullerene derivative, facilitating enhanced light absorption and charge separation.

In the case of the PDPP3T-PC_71_BM complex, the theoretical absorption spectrum shows a peak at 960 nm, which arises from a transition involving a partially occupied (*n*) molecular orbital to the π* state. This localized state, along with associated distortion, provides an easy pathway for electron/hole transport along the π-conjugated backbone, making this material particularly suited for applications in the Solar Cell.

**Figure 4:**
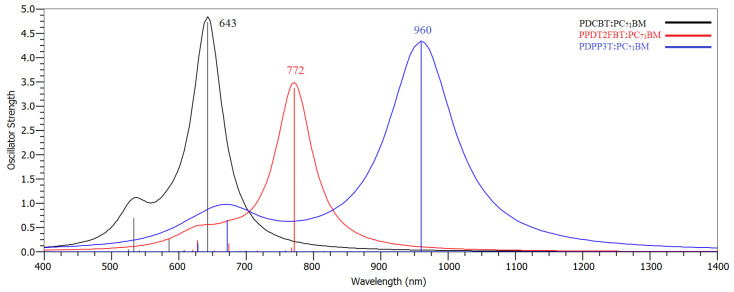
Simulated UV-Vis spectra of PDCBT-PC_71_BM, PPDT2FBT-PC_71_BM, and PDPP3T-PC_71_BM Complexes.

Hence, the absorption spectra of PDCBT, PPDT2FBT, and PDPP3T, both as pure polymers and in their PC_71_BM complexes, show excellent coverage of the solar spectrum from 350 to 1000 nm in Figure 4. These materials, particularly when paired with fullerene derivatives such as PC_71_BM, exhibit complementary absorption profiles, which are critical for efficient light harvesting in solar cells.

The theoretical spectra, while slightly red-shifted compared to experimental data, capture the essential absorption characteristics of the polymers. The discrepancies observed between the theory and experiment are primarily due to environmental effects, such as solvent interactions and solid-state packing, which are not fully accounted for in the gas-phase DFT calculations.

### 3.4. Molecular Electrostatic Potential (MEP) Surface Maps of PDCBT, PPDT2FBT, and PDPP3T

**Figure 5:**
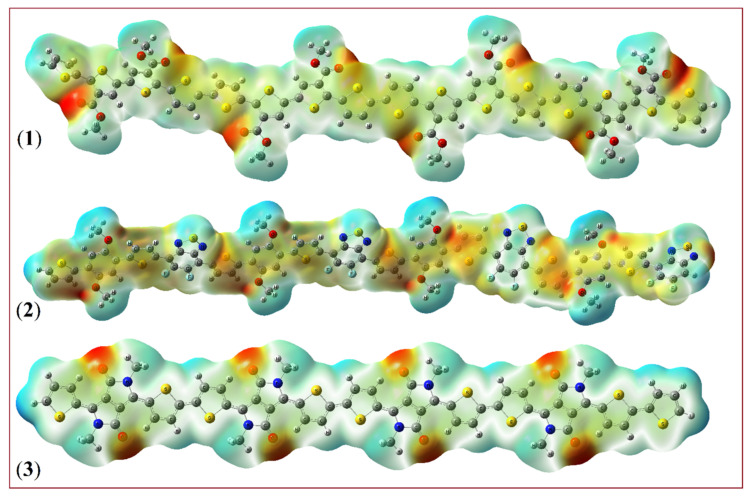
MEP plots of (**1**) PDCBT, (**2**) PPDT2FBT, and (**3**) PDPP3T polymer.

The reactivity and charge distribution in the polymers PDCBT, PPDT2FBT, and PDPP3T can be better understood through the analysis of their Molecular Electrostatic Potential (MEP) surface maps. These maps are critical for visualizing regions of electron density, which can indicate reactive sites for interactions in donor–acceptor complexes like polymer-PC_71_BM blends. The MEP surface map, shown in Figure 5, highlights areas of positive (blue), negative (red), and neutral (green) electrostatic potential across the molecular structure of each polymer. The minimum potential values (typically negative) correspond to regions where the electrostatic potential is lowest, often near electronegative atoms such as oxygen, nitrogen, or within π-electron-rich regions, and represent electrophilic attack sites. Neutral Zones are areas with minimal reactivity, typically appearing as green, representing regions of the molecule with balanced charge distributions. Herein, for these polymers, the maximum and minimum potential values are 0.0825 and −0.103 a.u.

**Figure 6:**
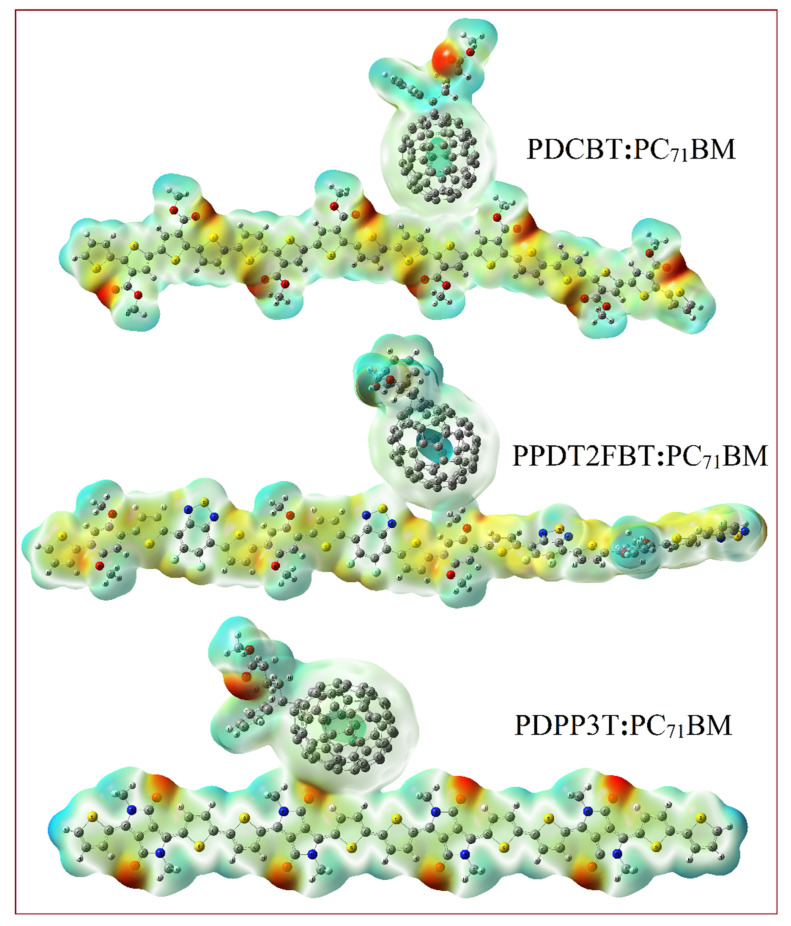
MEP plots of PDCBT-PC_71_BM, PPDT2FBT-PC_71_BM, and PDPP3T-PC_71_BM Complexes.

For the polymer-PC_71_BM complexes (PDCBT-PC_71_BM, PPDT2FBT-PC_71_BM, and PDPP3T-PC_71_BM), the MEP maps shown in Figure 6 further delineate the reactivity of these blends. The regions with the highest electron density (negative potential) are identified as key interaction sites where the polymers engage in electron transfers with the fullerene derivative PC_71_BM.

The MEP maps of the polymer-PC_71_BM complexes suggest that each polymer has distinct reactive sites that align with regions of a high electron density. This information is crucial for understanding the efficiency of these materials in the Solar Cell, as it informs how well each polymer can interact with the fullerene acceptor to facilitate electron transport.

Furthermore, the MEP surface maps offer valuable information on the charge distribution and reactive sites, which are critical for understanding and improving the performance of these materials in Solar Cell devices.

### 3.5. Molecular Orbital Study of PDCBT, PPDT2FBT, and PDPP3T Polymers and Their Complexes with PC71BM

Molecular orbital analysis is essential for understanding the electronic properties and behavior of materials, particularly in the design and computational modeling of sensors. For the polymers PDCBT, PPDT2FBT, and PDPP3T, the relative energies of HOMO and LUMO are key factors that dictate their reactivity and sensing capability.

**Figure 7:**
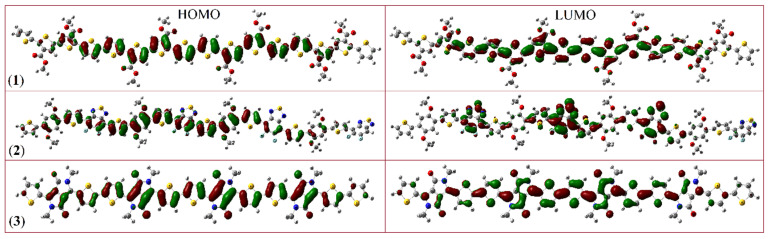
HOMO–LUMO plots of (**1**) PDCBT, (**2**) PPDT2FBT, and (**3**) PDPP3T polymer.

When these polymers interact with analytes, significant changes can occur in the HOMO and LUMO energy levels. For example, basic analytes tend to lower the excitation energy of the HOMO, while acidic analytes raise the excitation energy. This alteration in the HOMO–LUMO gap directly affects the electron transfer properties of the polymer, with HOMO acting as the electron donor (due to its electron-rich nature) and LUMO as the electron acceptor (due to its electron deficiency). The molecular orbital diagrams for PDCBT, PPDT2FBT, and PDPP3T are depicted in Figure 7. In Figure 7, (1) is the PDCBT polymer where the HOMO is delocalized across the entire polymer backbone, while the LUMO is similarly spread out, indicative of good electron mobility. Figure 7 (2) demonstrates that the PPDT2FBT HOMO is again distributed throughout the polymer, but the LUMO is more concentrated in the center, suggesting a different electronic interaction compared to the other two polymers. Whereas, in Figure 7, (3) is PDPP3T, representing the HOMO and LUMO as delocalized, similar to PDCBT, providing good electronic characteristics for charge transport.

These illustrations provide a visual understanding of the electron distribution across the polymer systems: The HOMO is spread across the entire polymer chain in all three polymers, which enhances their electron-donating capabilities. The LUMO is localized differently in the three polymers. For PDCBT and PDPP3T, the LUMO is distributed throughout the system, indicating a delocalized electron-accepting region. In contrast, for PPDT2FBT, the LUMO is more centralized within the polymer chain. These differences in the electron distribution affect the electrical properties of the polymers, particularly their band gap, which in turn influences their reactivity.

**Figure 8:**
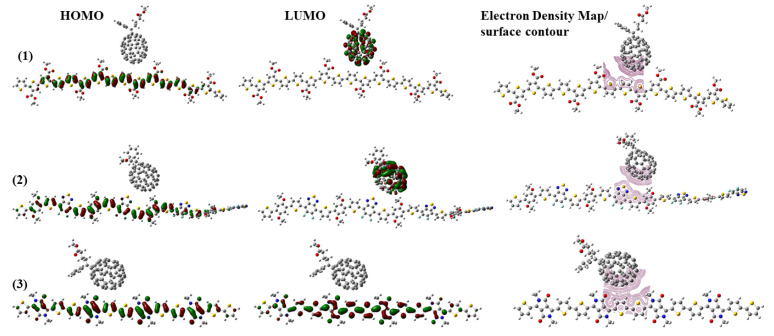
HOMO–LUMO plots and electron density maps of (**1**) PDCBT-PC_71_BM, (**2**) PPDT2FBT-PC_71_BM, and (**3**) PDPP3T-PC_71_BM complexes along with electron density map surface contour.

When the polymers are complexed with PC_71_BM, significant shifts are observed in the LUMO energy levels. The HOMO remains largely unchanged in these complexes, as shown in Figure 8.

For PDCBT-PC_71_BM and PPDT2FBT-PC_71_BM, the LUMO shifts completely toward the PC_71_BM acceptor, demonstrating that the complex facilitates an efficient electron transfer from the polymer to the fullerene. In the case of PDPP3T-PC_71_BM, however, the LUMO does not shift as dramatically, indicating a different interaction mechanism with PC_71_BM, potentially affecting the overall charge separation efficiency in photovoltaic applications.

These molecular orbital diagrams reveal the fundamental interaction between donor polymers and the fullerene acceptor, highlighting the efficiency of the electron transfer based on the relative positioning of the LUMO in the complex. Such analysis provides critical insights into the design of materials for Solar Cells and other electronic applications, where an efficient electron transfer and minimized recombination are essential for their performance. The molecular orbital study reveals essential characteristics of PDCBT, PPDT2FBT, and PDPP3T, particularly in relation to their HOMO–LUMO distributions and interactions with PC_71_BM in complexes.

The ability of these polymers to donate electrons, as well as their electronic and reactivity properties, can be understood by analyzing the energy band gaps and the HOMO–LUMO shifts in the complexes. Such insights are invaluable for optimizing these materials for solar cells and other electronic devices, where charge separation and transfer efficiency are paramount.

Table 1 shows the experimental and Density Functional Theory (DFT) calculated values for the Highest HOMO, LUMO, and band gap for three polymers: PDCBT, PPDT2FBT, and PDPP3T. These values are presented for varying numbers of repeating units, from 1 to ∞, where ∞ represents the estimated properties of the infinite polymer, typically extrapolated using a second-order polynomial fit. Triple-junction solar cells benefit from using multiple materials that can absorb light at different parts of the solar spectrum. Each polymer in the table has distinct HOMO, LUMO, and band gap values, which influence their role in a tandem cell. As the Experimental Band Gap (1.90 eV) and DFT-calculated Band Gap for repeating units increase, the band gap gradually decreases from 3.19 eV (1 unit) to 2.14 eV (∞ units) in PDCBT. Hence, relevant for Solar Cells, the large band gap of PDCBT indicates it can absorb higher-energy photons, typically from the UV part of the spectrum. As the number of repeating units increases, the band gap decreases, which brings it closer to the experimental value, reflecting how long chains of PDCBT would behave in a real-world scenario. The Experimental Band Gap (1.76 eV) and DFT-calculated band gap decreases from 2.55 eV (1 unit) to 1.84 eV (∞ units) for PPDT2FBT, aligning closely with the experimental value as the polymer length increases as PPDT2FBT absorbs the lower-energy part of the visible spectrum compared to PDCBT. Its band gap is smaller, meaning it will be ideal for capturing photons in the middle part of the solar spectrum. Whereas, for the PDPP3T experimental band gap (1.56 eV) and DFT-calculated band gap, that start from 2.28 eV (1 unit), the band gap drops significantly to 1.41 eV for the infinite polymer, which is close to the experimental value. PDPP3T in solar cells, due to the smallest band gap of the three polymers, can absorb light in the red and near-infrared parts of the solar spectrum, making it ideal for the lower energy section in a triple junction solar cell, ensuring the efficient use of the solar spectrum. The DFT-calculated band gaps for the infinite polymers approach the experimental values, which indicates that these theoretical calculations are reliable for understanding how long polymer chains behave in real-world applications. For all three polymers, the HOMO levels become less negative (closer to zero) as the polymer chain length increases, reflecting the stabilization of the frontier orbitals as the polymer grows longer. This is an essential factor in optimizing electron transfer processes in solar cells. So far, Table 1 offers essential data for selecting and positioning materials in a triple-junction solar cell, where the polymers’ differing band gaps allow for a complementary absorption of the solar spectrum, maximizing the cell’s efficiency.

### 3.6. Non-Covalent Interactions of PDCBT-PC_71_BM, PPDT2FBT-PC_71_BM, and PDPP3T-PC_71_BM Complexes

This study focuses on the non-covalent interactions of the polymers PDCBT, PPDT2FBT, and PDPP3T with PC_71_BM, explaining the significance of bond distances for sensor and electron transfer efficiency. The non-covalent interactions between these polymers and PC_71_BM is crucial for evaluating their sensing capabilities and overall performance. Figure S1 presents the optimized geometrical structures of the polymer-PC_71_BM complexes, showcasing the interaction distances between various atoms. For the PDCBT-PC_71_BM complex, several key interactions observed that Thiophene hydrogen atoms in PDCBT form bonds with PC_71_BM at distances of 3.85 Å, 3.03 Å, and 4.15 Å. These bond distances indicate a moderate-to-close interaction between the polymer and the fullerene (PC_71_BM). The methyl hydrogen in PDCBT shows a bond distance of 3.0 Å with PC_71_BM, indicating a relatively strong interaction. The sulfur atom in PDCBT interacts with PC_71_BM at a bond distance of 4.58 Å, which is slightly weaker than the hydrogen–carbon interactions, but still significant for charge transfer processes. These interactions demonstrate that the PDCBT polymer establishes multiple binding points with PC_71_BM, facilitating an electron transfer and enabling its role in sensing and photovoltaic applications. In the PPDT2FBT-PC_71_BM complex, the interaction between the polymer and PC_71_BM involves several atoms like sulfur atoms in PPDT2FBT, which form a bond with PC_71_BM at a distance of 4.06 Å, which is weaker than the sulfur–carbon interaction in the PDCBT complex. The nitrogen atom in PPDT2FBT shows a bond distance of 4.80 Å with PC_71_BM, indicating a comparatively weaker interaction compared to sulfur. The methyl hydrogen atoms have bond distances of 3.18 Å and 3.46 Å with PC_71_BM, highlighting stronger non-covalent interactions than the sulfur and nitrogen atoms. Overall, the PPDT2FBT-PC_71_BM complex exhibits a combination of moderately strong and weak interactions, with the methyl hydrogen providing the strongest binding points. This suggests that PPDT2FBT can also facilitate the charge transfer, although the interactions may not be as strong as in PDCBT. The PDPP3T-PC_71_BM complex has the closest interactions among the three polymer complexes. The thiophene hydrogen atoms in PDPP3T form bonds with PC_71_BM at distances of 3.09 Å and 3.18 Å, indicating very close interactions that can facilitate an effective electron transfer. The methyl hydrogen atoms interact with PC_71_BM at a bond distance of 3.28 Å, which is similar to the thiophene hydrogen-PC_71_BM interaction. The oxygen atom in PDPP3T shows a bond distance of 3.42 Å with PC_71_BM, indicating moderate interaction strength. These close interaction distances, particularly the strong thiophene hydrogen and methyl hydrogen bonds, suggest that PDPP3T is well-suited for applications requiring an efficient charge transfer. The closer interaction distances in the PDPP3T-PC_71_BM complex make it highly effective in sensing applications, where a rapid electron transfer is essential. In short, the sensor study of the PDCBT-PC_71_BM, PPDT2FBT-PC_71_BM, and PDPP3T-PC_71_BM complexes reveals varying degrees of non-covalent interactions between the polymers and PC_71_BM. These interactions play a vital role in determining the sensing capabilities and electron transfer efficiency of the complexes. These findings highlight the importance of molecular interaction distances in optimizing the performance of polymer-PC_71_BM complexes in sensor applications, particularly for electronic sensing and photovoltaic devices.

### 3.7. Theoretical Performance of Polymer Solar Cells

The triple-junction structure is designed with three different photoactive materials, each targeting a specific portion of the solar spectrum. The front junction (1.9 eV) absorbs higher-energy photons (blue/UV light). The middle junction (1.63 eV) absorbs mid-energy photons. The back junction (1.33 eV) absorbs lower-energy photons (infrared). The combination of these three junctions maximizes the absorption of solar energy, leading to higher efficiency compared to single- or double-junction solar cells.

**Figure 9:**
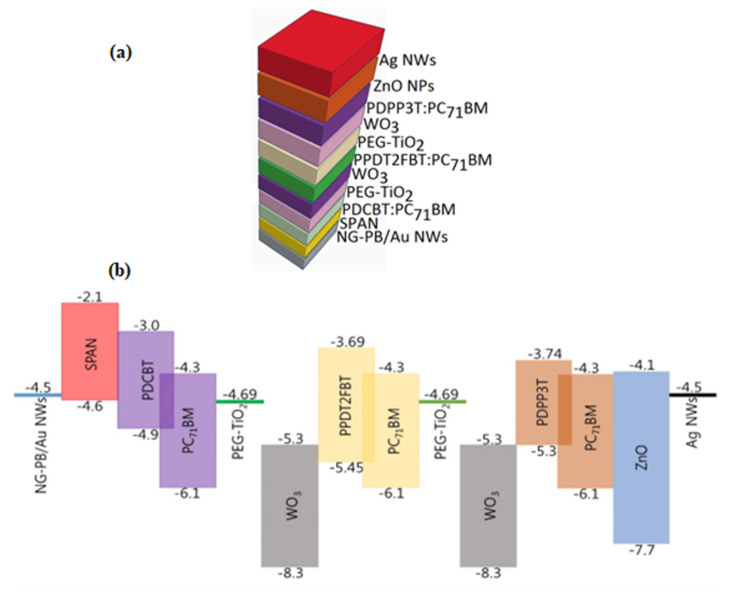
(**a**) Depicts the device structure of a conventional triple-junction solar cell, showing how different materials are stacked in the design to achieve high efficiency. (**b**) Displays the energy diagram of the conventional triple-junction solar cell, illustrating the energy levels of each layer and how charge carriers (electrons and holes) move through the system.

A triple-junction solar cell composed of multiple layers, with each junction optimized for a different part of the solar spectrum, is shown in Figure 9a,b. These layers help in achieving high efficiency by utilizing a broader range of the solar spectrum. Here is a breakdown of the structure. The Top Layer is Ag NWs (Silver Nanowires) which acts as a transparent conductive electrode, allowing light to enter while maintaining electrical conductivity. ZnO NPs (Zinc Oxide Nanoparticles) act as an electron transport layer, facilitating electron movement while blocking holes. The Back Junction has a Band Gap Eg = 1.33 eV: PDPP3T:PC71BM. This layer consists of a polymer donor (PDPP3T) and a fullerene acceptor (PC71BM) blend, which forms the photoactive layer responsible for absorbing light and generating excitons (electron–hole pairs). While the Middle Junction (Band Gap Eg = 1.63 eV: WO_3_ (Tungsten Trioxide) is a buffer layer that helps in the movement of holes and blocks electrons, which improves the charge separation and prevents recombination. PEG-TiO_2_ (Polyethylene Glycol with Titanium Dioxide) is a modified electron transport layer that enhances electron mobility. PPDT2FBT:PC_71_BM is another polymer-fullerene blend layer that absorbs light in the middle of the solar spectrum. The Front Junction, having Band Gap Eg = 1.9 eV: WO_3_ and PEG-TiO_2_ layers, is similar to the middle junction, and these layers act as hole and electron transport layers. PDCBT:PC_71_BM is the photoactive material in this layer, absorbing light in the high-energy (blue/UV) portion of the spectrum. SPAN is the substrate and the Bottom Layers are an additional layer used for hole transport. NG-PB/Au NWs (Nanographene-Polyaniline/Gold Nanowires) are part of the electrode system, where NG and Au NWs help in conducting electricity.

### 3.8. Electronic Properties of Triple-Junction Solar Cell Materials

The cell is constructed with three sub-cells, each made of a different combination of donor and acceptor materials, which have different band gaps. In this case: PDCBT:PC_71_BM (Front junction), PPDT2FBT:PC71BM (Middle junction), and PDPP3T:PC71BM (Back junction)

**Figure 10:**
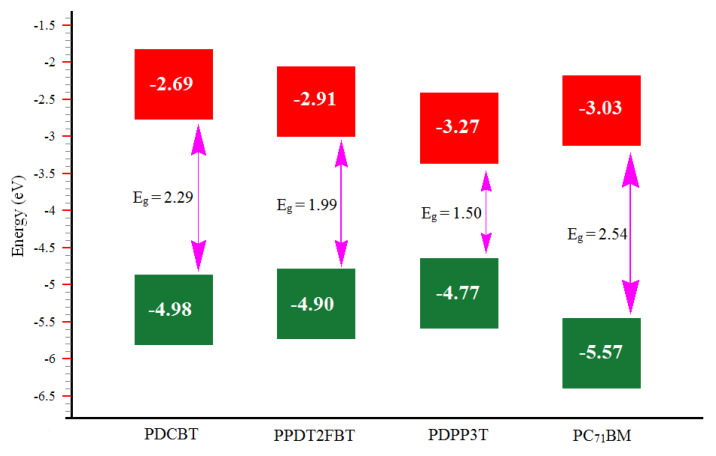
Energy level diagram of PDPP3T, PPDT2FBT, PDPP3T, and PC_71_BM system.

Band gaps computed at DFT level are typically underestimated in Table 1. Each donor material (PDCBT, PPDT2FBT, and PDPP3T) has a unique band gap, ensuring that the solar cell captures photons from a broader energy range: PDCBT has a band gap of 2.29 eV. PPDT2FBT has a band gap of 1.99 eV. PDPP3T has a band gap of 1.50 eV. These band gaps are combined with PC_71_BM, which has a LUMO of −3.03 eV and a band gap of 2.54 eV shown in Figure 10.

The energy levels of the HOMO and LUMO for each donor material and the acceptor (PC_71_BM) are carefully aligned to facilitate efficient charge separation. Figure S2 presents PDCBT, which has a HOMO of −4.98 eV and a LUMO of −2.69 eV, while PPDT2FBT has a HOMO of −4.90 eV and a LUMO of −2.91 eV, as shown in Figure S3 and Table S1. By stacking the sub-cells in layers, the triple-junction structure ensures that each layer absorbs a specific portion of the solar spectrum, thus improving the overall efficiency. The different band gaps allow for the optimal utilization of low, medium, and high-energy photons, thereby reducing energy losses and improving the photogenerated current.

The exciton binding energy (Eb) for the donor–acceptor combinations is another critical factor. PDCBT has an exciton binding energy of 0.39 eV, while PPDT2FBT has 0.35 eV, as shown in Table 1. Hence, the triple-junction solar cell combines layers of materials like PDCBT, PPDT2FBT, and PDPP3T with PC_71_BM to enhance solar energy absorption across a wide range of wavelengths. The careful alignment of energy levels and band gaps leads to higher efficiencies compared to single-junction cells.

## 4. Conclusions

This research explores advanced polymer solar cells using PDCBT, PPDT2FBT, and PDPP3T as donor materials with PC71BM as an electron acceptor. DFT and TD-DFT simulations reveal excellent molecular geometries, broad UV-Vis absorption, and efficient charge transport. These findings highlight the potential of these materials for high-performance solar cells and pave the way for future innovations in multi-junction solar technology.

## Figures and Tables

**Table 1:** HOMO, LUMO, Band gap, Optical Gap, Photovoltaic Energy Gap (E_PVG_), and LUMO^Donor^–LUMO^Acceptor^ Exciton Binding Energy (E_b_) of PC_71_BM, Polymers and PC_71_BM-Bounded Polymers.

Species	HOMO	LUMO	Band Gap	Optical Gap	λ	E_PVG_	E_b_
PC_71_BM	−5.57	−3.03	2.54	2.33	0.120		0.21
PDCBT	−4.98	−2.69	2.29	1.90	0.155		0.39
PPDT2FBT	−4.90	−2.91	1.99	1.64	0.122		0.35
PDPP3T	−4.77	−3.27	1.50	1.25	0.155		0.25
PDCBT:PC_71_BM	−4.97	−3.06	1.91	1.93		1.95	
PPDT2FBT:PC_71_BM	−5.0	−3.17	1.83	1.60		1.87	
PDPP3T:PC_71_BM	−4.76	−3.27	1.49	1.29		1.74	

## Data Availability

The original contributions presented in the study are included in the article and Supplementary Materials; further enquiries can be directed to the corresponding authors.

## Supplementary Materials

The following supporting information can be downloaded at: https://www.mdpi.com/article/10.3390/molecules29225370/s1.
