# Supplementary material for: High-Efficiency Triple-Junction Polymer Solar Cell: A Theoretical Approach
Source: Molecules. 2024 Nov 14;29(22):5370. doi: 10.3390/molecules29225370 (PMC11596838; doi:10.3390/molecules29225370)
Supplement: Supplementary file 1 [file molecules-29-05370-s001.zip › molecules-3270811-supplementary.pdf]

High-Efficiency Triple-Junction Polymer Solar Cell: A Theoretical Approach

Fazli Sattar[1], Xiaozhuang Zhou[1*], and Zakir Ullah[2*]

[1] Yangtze Delta Region Institute (Huzhou), University of Electronic Science and Technology of China, Huzhou, 313001. P. R. China.

[2] Institut de Ciència de Materials de Barcelona (ICMAB-CSIC), Consejo Superior de Investigaciones Científicas, Campus Universitari de Bellaterra, Cerdanyola del Vallès 08193, Spain.

* Correspondence: zhouxz@csj.uestc.edu.cn (X.Z.); azazay1@gmail.com (Z.U.)

ABSTRACT: This study presents the theoretical design and evaluation of a triple-junction polymer solar cell architecture, incorporating oligomers of PDCBT, PPDT2FBT, and PDPP3T as donor materials and $PC_{71}BM$ as the electron acceptor. Using density functional theory (DFT) simulations and time-dependent DFT (TD-DFT) methods, the investigation covers essential photovoltaic parameters, including molecular geometries, UV-Vis spectra, and charge transport properties. The device is structured to maximize solar energy absorption across the spectrum, featuring front, middle, and back junctions with band gaps of 1.9 eV, 1.63 eV, and 1.33 eV, respectively. Each layer targets different regions of the solar spectrum, optimizing light harvesting and charge separation. This innovative multi-junction design offers a promising pathway to enhanced power conversion efficiencies in polymer solar cells, advancing the integration of renewable energy technologies.

Keywords: Solar Cell, DFT, TD-DFT, UV-Vis, HOMO, LUMO

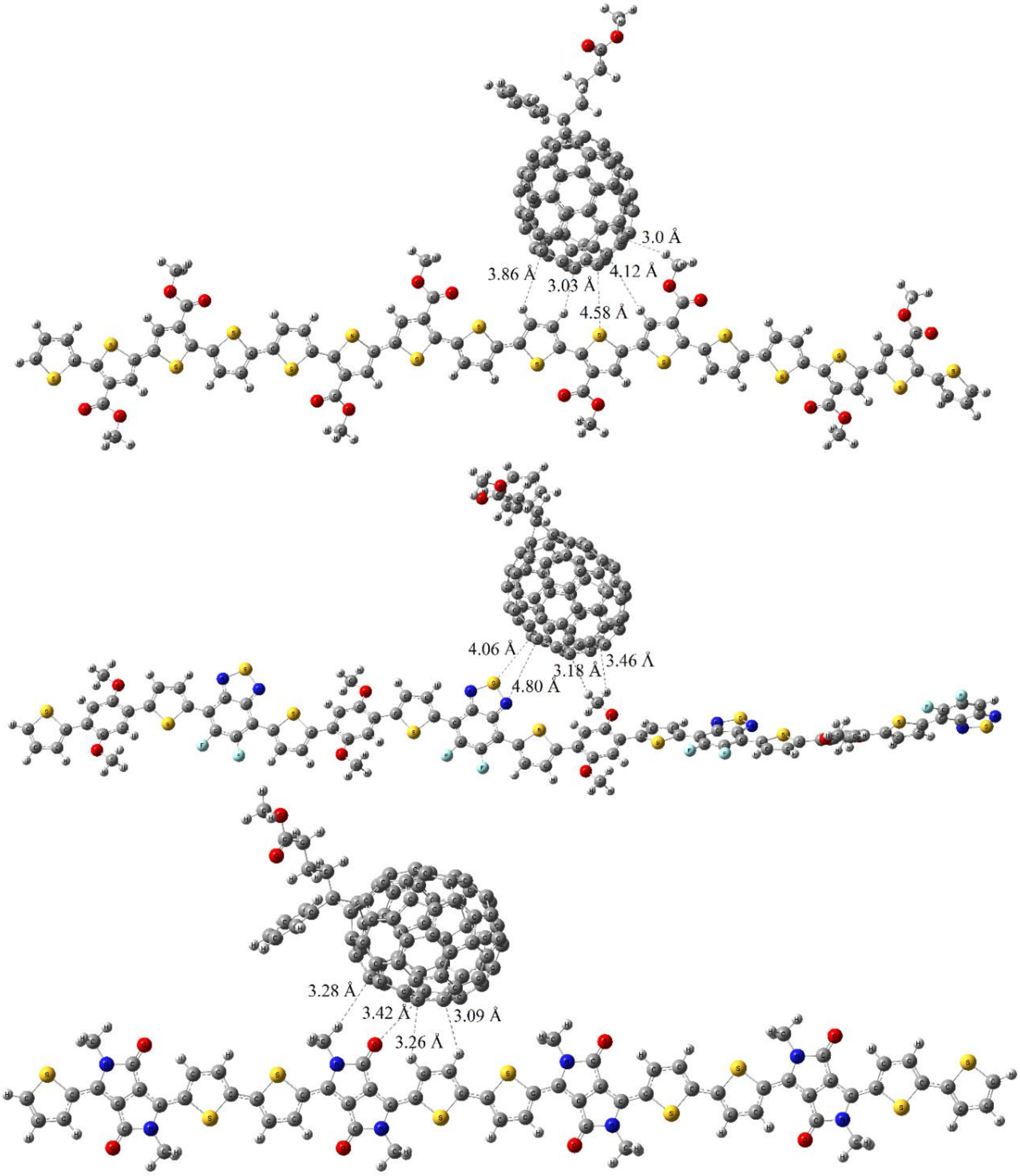

Figure S1. Optimized Geometric Structures of PDCBT-, PPDT2FBT-, and PDPP3T-$PC_{71}BM$ systems

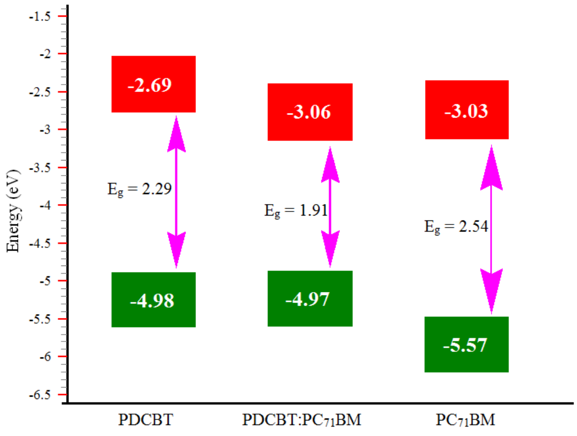

Figure S2. Energy level diagram of PDCBT, $PC_{71}BM$, and PDCBT-PC71BM system

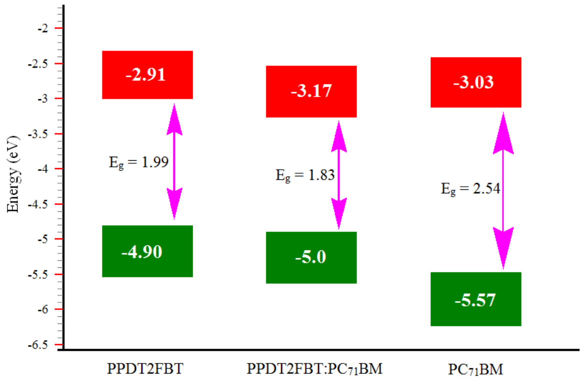

Figure S3. Energy level diagram of PPDT2FBT, $PC_{71}BM$, and PPDT2FBT-$PC_{71}BM$ system

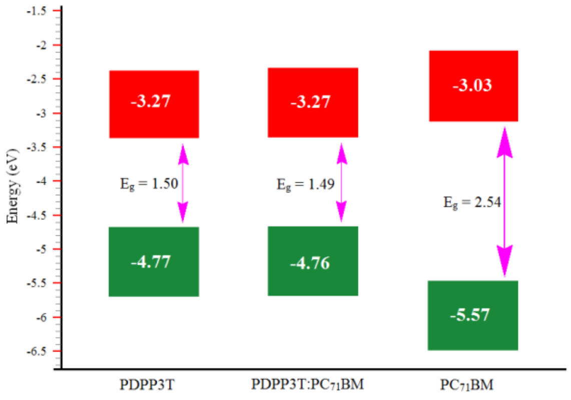

Figure S4. Energy level diagram of PDPP3T, $PC_{71}BM$, and PDPP3T-PC71BM system

Table S1. Experimental and DFT calculated HOMO, LUMO, and band gap of PDCBT, PPDT2FBT, and PDPP3T polymer; the infinite polymer properties are estimated from the second order polynomial fit equation.

Species	*No of Rep unit*	*HOMO*	*LUMO*	*Band gap*
1-PDCBT	Experimental	-4.9	-3.0	1.90
	1PDCBT	-5.39	-2.20	3.19
	2PDCBT	-5.08	-2.57	2.51
	3PDCBT	-5.0	-2.66	2.34
	4PDCBT	-4.98	-2.69	2.29
	∞**PDCBT**	**-4.91**	**-2.77**	**2.14**
2-PPDT2FBT	Experimental	-5.45	-3.69	1.76
	1PPDT2FBT	-5.20	-2.65	2.55
	2PPDT2FBT	-4.99	-2.83	2.16
	3PPDT2FBT	-4.93	-2.88	2.05
	4PPDT2FBT	-4.90	-2.91	1.99
	∞**PPDT2FBT**	**-4.82**	**-2.98**	**1.84**
3-PDPP3T	Experimental	-5.30	-3.74	1.56
	1PDPP3T	-4.99	-2.71	2.28
	2PDPP3T	-4.85	-3.06	1.79
	3PDPP3T	-4.80	-3.20	1.60
	4PDPP3T	-4.77	-3.27	1.50
	∞**PDPP3T**	**-4.70**	**-3.29**	**1.41**
